# Talon Noir: A Case Report and Literature Review

**DOI:** 10.7759/cureus.35905

**Published:** 2023-03-08

**Authors:** Sampa Choudhury, Ashish Mandal

**Affiliations:** 1 Department of Pathology, Andaman & Nicobar Islands Institute of Medical Sciences, Port Blair, IND; 2 Department of Pathology, Laboratory Medicine and Blood Bank, School of Medical Sciences and Research, Sharda University, Greater Noida, IND

**Keywords:** talon noir, shearing force, melanoma, dermoscopy, black heel

## Abstract

Talon noir is an asymptomatic, self-limiting, dermatological condition that is most commonly seen in young athletes and mountain climbers. Histologically, the characteristic findings are intraepidermal hemorrhage and fluid collection due to shearing forces. Here, we report an interesting case in an elderly female, which mimicked acral melanoma clinically and alerted us to do a biopsy for confirmation.

## Introduction

Talon noir or black heel is a dermatological condition characterized by intraepidermal hemorrhage and exudation due to shearing forces in an area of recurrent or sudden trauma [1]. It is commonly seen in young athletes involving the heels and palms [2,3]. This benign, self-limiting condition can be mistaken as melanoma and should be differentiated from the latter because of therapeutic and prognostic differences [3,4].

In our case, we report a case of talon noir in a middle-aged social activist woman with no history of sports activity.

## Case presentation

**Figure 1:**
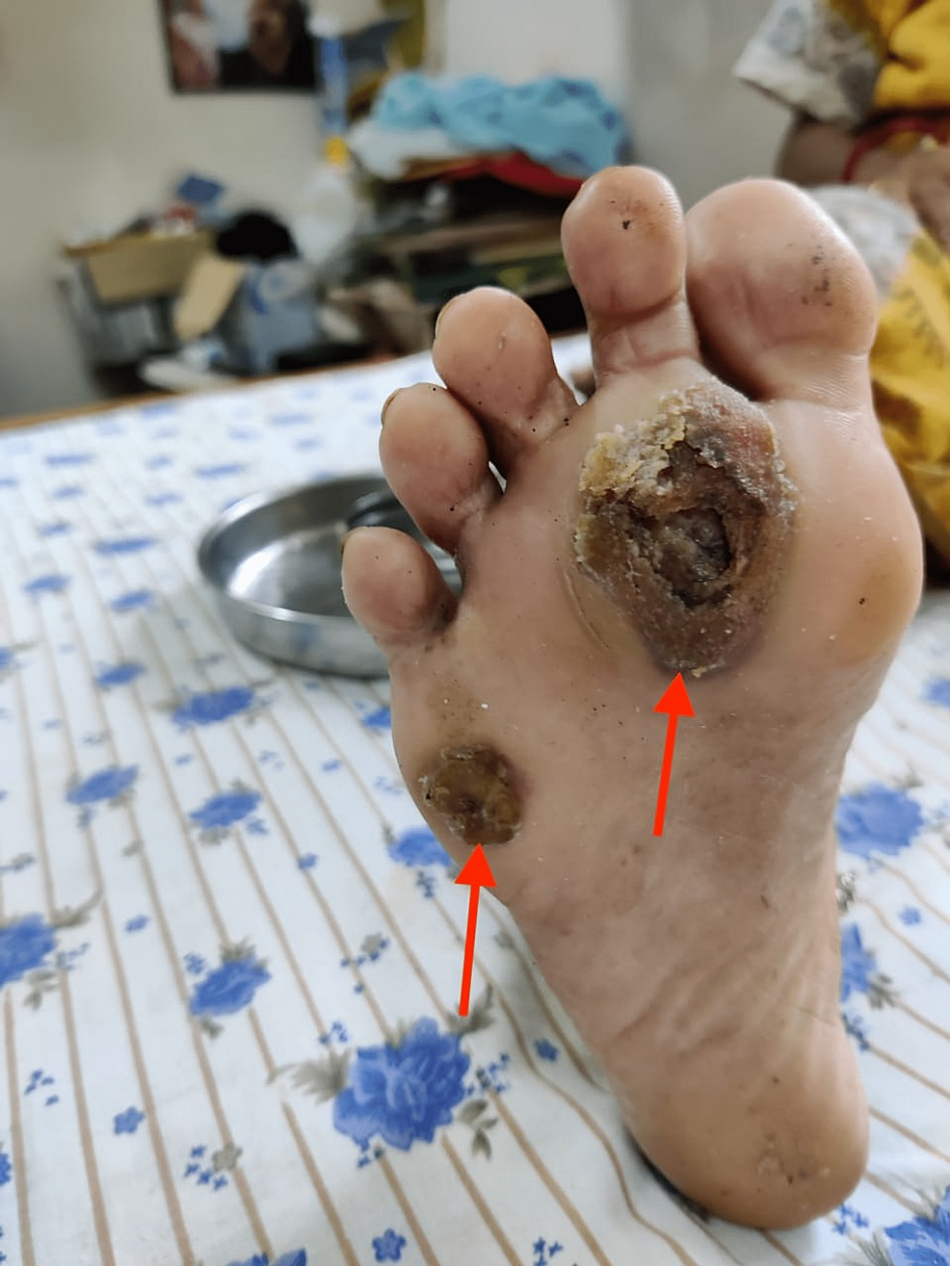
Two irregular, blackish-brown plaques on the plantar aspect of the right foot (red arrows).

**Figure 2:**
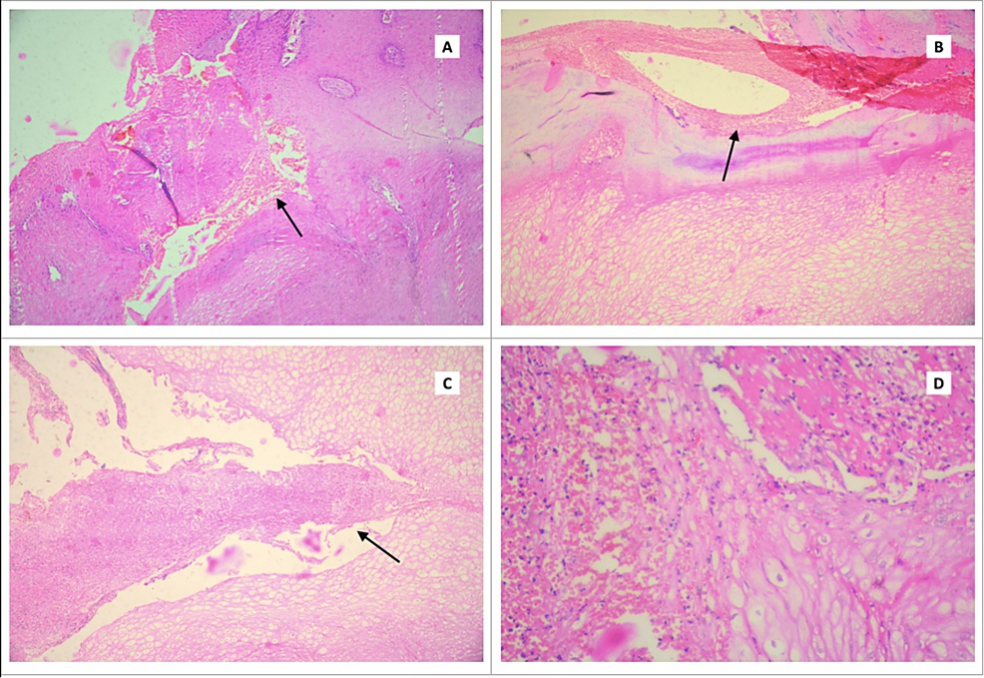
Histopathological findings from the blackish-brown plaques of the foot showing a benign lesion due to repeated microtrauma. A and B: Intraepidermal collections of degenerated red cells (black arrow, hematoxylin and eosin, 20×). C: Focal acute inflammatory exudate (black arrow, hematoxylin and eosin, 20×). D: Intraepidermal inflammatory exudate (hematoxylin and eosin, 40×).

A 49-year-old diabetic female, a radio announcer and social activist by profession, presented with two dark blackish-colored plaques on her right sole for one year. These lesions were painless and gradually increased in size. There was no history of injury or trauma. However, the patient provided a history of using high-heeled shoes and walking long distances for work. On examination, two irregular, thickened, blackish-brown plaques were identified on the plantar aspect of the right forefoot measuring 4.5 × 3 cm and 1.5 × 1.5 cm, respectively (Figure 1). The left foot was normal. All other general physical and systemic examinations were within normal limits. Her random blood sugar and hemoglobin A1C levels were 290 mg/dL and 9.1%, respectively. Other hematological investigations were within normal limits. Dermoscopy could not be performed because of its unavailability in our institute. Hence, a clinical diagnosis of melanoma was entertained and an excision biopsy was performed. Microscopic examination showed severe hyperkeratosis, parakeratosis, and acanthosis. Multiple foci of acute inflammatory exudates and degenerated red cells were seen in the epidermis, while dermal papillae showed large areas of hemorrhage (Figure 2). There was no evidence of melanoma. A final diagnosis of talon noir or black heel was made. There was no recurrence of any lesions on follow-up visits.

## Discussion

Talon noir was first described in 1961 as asymptomatic, bilateral, deep-seated petechiae on posterolateral aspects of the heels of basketball players [2]. There are many synonyms for this condition, such as black heel, calcaneal petechiae, subcorneal hematoma, intracorneal hematoma, and hyperkeratosis hemorrhagica, among others [5].

We searched the literature on PubMed and Google Scholar using the keywords “Talon noir,” “Black heel,” and “Subcorneal hematoma” from 2012 to 2021 and retrieved 14 articles with full text or abstract available reporting 51 such cases in English literature. Most cases were reported involving the heel, sole of the foot, toes, and palm of adolescents and young adults with a history of sports activities, repeated trauma, mountain climbing, or taking anticoagulant therapy. A few cases were also observed involving extremities of age with no such typical histories. An unusual case of a viral infection-associated black heel has also been reported [4,6,7].

Clinically, it presents as asymptomatic, asymmetrical, black-to-brown-colored macules, isolated or in clusters, with an uninterrupted normal skin surface [3]. The pathogenesis can be explained by damage of the papillary dermal blood vessels due to shearing forces related to sports activity, climbing, or any kind of repetitive microtrauma that eventually leads to the leakage of blood from the dermis to the epidermis [3,8]. In our case, the lesions had an unusual location on the forefoot just below the metatarsals, and there was no association with sports activity. However, uncomfortable, ill-fitting shoes, walking long distances, and uncontrolled diabetes may be the reasons for the repeated injuries of the sole and delayed healing which lead to the development of these pigmented lesions. However, as no study was found to support the association of talon noir with diabetes mellitus, further exploration is mandated.

Differential diagnoses usually include acral melanoma, acral nevi, plantar wart, pyogenic granuloma, angiokeratoma, corn, and tinea nigra [1,5]. A wart has a verrucous surface with disrupted skin lines. Tinea nigra, an unusual fungal infection, can present as a brownish macule due to melanin-like pigment production. This condition can be easily differentiated from talon noir by a potassium hydroxide scraping test which highlights its hyphae and spores [3,9].

Dermoscopy, a non-invasive procedure, can differentiate talon noir from melanoma preoperatively as the former shows homogenous reddish globular structures [10]. Acral melanoma, on the contrary, shows a typical parallel ridge pattern with irregular pigmentation. Because some studies have claimed that dermoscopic features may be misleading, scraping test and biopsy are the preferred methods to differentiate between these two conditions, especially long-standing cases with dubious findings [4,7,8]. Scraping or paring down of talon noir leads to partial or complete loss of pigmentation, whereas melanocytic lesions remain the same [9,11,12].

It is a self-limiting benign condition that usually resolves within a few weeks by avoiding sports activity or any kind of repetitive trauma and using well-cushioned shoes, thick socks, or skin lubrication. Exanthema-associated capillary fragility can be benefited from vitamin C supplementation [6,11-14]. In our case, because of the unusual localization and ambiguity in history, acral melanoma was suspected, and characteristic histopathological findings of the excision biopsy specimen confirmed the diagnosis as talon noir.

Table 1 presents the cases of talon noir or black heel reported from 2012 to 2021 in the English-language literature.

**Table 1:** Cases of talon noir or black heel reported from 2012 to 2021 in the literature. #: Age and gender are not available in the abstract. F = female; M = male; NA = not available

Author name	Number of cases	Patient(s) age (years)	Gender	Occupation/Associated history	Location	Pattern of lesion	Dermoscopy findings	Treatment
Nassar et al., 2012 [1]	18	32 ± 17.5 (mean)	14 M, 4 F	History of trauma (3) and playing football (7). No remarkable history (8).	The sole of the foot (17) and palm (1)	Lesions of varying sizes, shapes, and colors. Black in eight (44.4%), red-black in six (33.3%), and brown in the remaining four (22.2%) lesions. Eight lesions (44.4%) had surrounding dark dots (satellites)	Homogeneous in 55.6%, globular in 44.4%, parallel ridge in 27.8%, and negative pseudo-network in the remaining 16.6% of lesions	Paring was performed by a dermatologist for 17 lesions. The patient’s fingernail scratching in one case led to the complete removal of pigmentation
Martin et al., 2021 [13]	1	Young#	NA	Baseball player	Plantar aspect of the foot	Pigmented macules	NA	Spontaneous resolution after 2-3 weeks
Khurana, 2020 [4]	1	75	F	Housewife, diabetes under control, and no history of trauma, fever, or any systemic illness	Over the right heel	Reddish brown-to-dark black macules	Homogenous globular pattern	The paring of the lesion
Goggins et al., 2020 [3]	1	16	M	Hockey and baseball player	Palm	Brown-black punctate macules in the center of hypothecate callus	Parallel ridge pattern	The paring of the lesion
Elmas and Akdeniz, 2019 [5]	20	41.4 (mean)	14 M, 6 F	Four had a history of trauma, and two had a history of anticoagulant therapy	60% soles, 25% palms, and 15% volar surface of the hand	Red-black-colored lesions followed by brown to black	Homogenous pattern in 65% globular pattern in 55% and parallel ridge pattern in 40%	Scratch test performed with an appropriate size of scalpel which allows complete clearance of the pigmentation
Tammaro et al., 2018 [8]	1	23	M	Climbing instructor	Heel, palmar arch, and medial border of the foot	Pigmented macules	Homogenous pattern	NA
Uslu et al., 2017 [7]	1	67	F	No history of trauma, new shoe, blister, and physical exercise	Plantar aspect of the right toe	Irregular brownish macule	Parallel ridge pattern	Excision
Jenna et al., 2016 [9]	1	13	M	Basketball practice	Posterior edge of the plantar surface of the heel	Violaceous-to-black, punctate macules	NA	NA
Keerthi et al., 2015 [2]	1	45	F	Farmer, no history of trauma, sports participation, or taking anticoagulant medicines	Tip of the first and second phalanges of the left foot	Black to light brown large patches	NA	Excision
Googe et al., 2014 [12]	1	34	F	No history of trauma, sports participation, or ill-fitting shoes. No history of taking anticoagulant drugs	Calcaneal surface of the left foot	Black brown macule	NA	The paring of the lesion
Brzezinski et al., 2014 [11]	2	16	M	Dancer	Outer border of the right and left heels	Speckled bluish-black macules	NA	Spontaneous resolution after a few weeks of rest
		28	M	Mountain climber (alpinist)	Outer surface of the right heel and the other one on the inner surface of the left heel	Speckled bluish-black macules		
Lao et al., 2013 [14]	1	15	M	Hockey and basketball player	Palm	Dark spot	NA	NA
Sardana et al., 2013 [6]	1	6	NA	Associated with viral exanthem	Sole of the foot	Pigmented macule	NA	Vitamin C supplementation for 7 days
Sharma et al., 2012 [10]	1	18	M	NA	Sole of the foot and at the base of the metatarsal area	Pigmented macule	NA	NA
Our case	1	49	F	History of high-heeled shoes, long-distance walking, and uncontrolled diabetes	Plantar aspect of the right forefoot	Two irregular, thickened, brownish-black plaques	Not done	Excision

## Conclusions

The present case mimicked melanoma due to its dark blackish color and irregular ulcerated surface. However, awareness of this entity could have saved the pain of excisional biopsy and the associated psychological trauma of being diagnosed with cancerous growth albeit provisionally. The importance of detailed history particularly of sports or long-distance trekking cannot be over-emphasized. This case may be a complication of diabetes; nonetheless, future studies are mandated due to the paucity of supportive literature. Thus, pigmented lesions are not always worrisome melanocytic lesions.
